# From Precision Colloidal Hybrid Materials to Advanced Functional Assemblies

**DOI:** 10.1021/acs.accounts.2c00093

**Published:** 2022-06-01

**Authors:** Veikko Linko, Hang Zhang, Nonappa, Mauri A. Kostiainen, Olli Ikkala

**Affiliations:** † Department of Bioproducts and Biosystems, Aalto University School of Chemical Engineering, FI-00076 Espoo, Finland; ‡ Department of Applied Physics, Aalto University School of Science, FI-00076 Espoo, Finland; ⊥ Faculty of Engineering and Natural Sciences, Tampere University, P.O. Box 541, FI-33101 Tampere, Finland

## Abstract

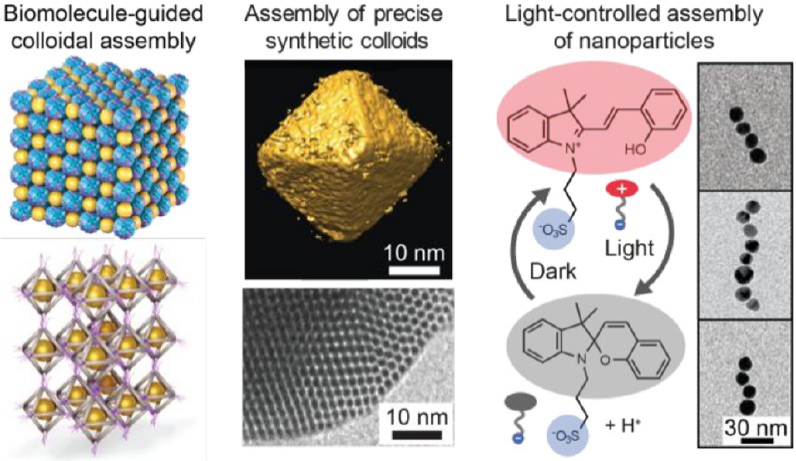

The concept of colloids encompasses a wide range of isotropic and anisotropic particles with diverse sizes, shapes, and functions from synthetic nanoparticles, nanorods, and nanosheets to functional biological units. They are addressed in materials science for various functions, while they are ubiquitous in the biological world for multiple functions. A large variety of synthetic colloids have been researched due to their scientific and technological importance; still they characteristically suffer from finite size distributions, imperfect shapes and interactions, and not fully engineered functions. This contrasts with biological colloids that offer precision in their size, shape, and functionality. Materials science has searched for inspiration from the biological world to allow structural control by self-assembly and hierarchy and to identify novel routes for combinations of functions in bio-inspiration.

Herein, we first discuss different approaches for highly defined structural control of technically relevant synthetic colloids based on guided assemblies of biological motifs. First, we describe how polydisperse nanoparticles can be assembled within hollow protein cages to allow well-defined assemblies and hierarchical packings. Another approach relies on DNA nanotechnology-based assemblies, where engineered DNA structures allow programmed assembly. Then we will discuss synthetic colloids that have either particularly narrow size dispersity or even atomically precise structures for new assemblies and potential functions. Such colloids can have well-defined packings for membranes allowing high modulus. They can be switchable using light-responsive moieties, and they can initiate packing of larger assemblies of different geometrical shapes. The emphasis is on atomically defined nanoclusters that allow well-defined assemblies by supramolecular interactions, such as directional hydrogen bonding. Finally, we will discuss stimulus-responsive colloids for new functions, even toward complex responsive functions inspired by life. Therein, stimulus-responsive materials inspired by biological learning could allow the next generation of such materials. Classical conditioning is among the simplest biological learning concepts, requiring two stimuli and triggerable memory. Therein we use thermoresponsive hydrogels with plasmonic gold nanoparticles and a spiropyran photoacid as a model. Heating is the unconditioned stimulus leading to melting of the thermoresponsive gel, whereas light (at a specified wavelength) originally leads to reduced pH without plasmonic or structural changes because of steric gel stabilization. Under heat-induced gel melting, light results in pH-decrease and chain-like aggregation of the gold nanoparticles, allowing a new plasmonic response. Thus, simultaneous heating and light irradiation allow conditioning for a newly derived stimulus, where the logic diagram is analogous to Pavlovian conditioning. The shown assemblies demonstrate the different functionalities achievable using colloids when the sizes and the dispersity are controlled.

## Key References

- KostiainenM. A.; HiekkataipaleP.; LaihoA.; LemieuxV.; SeitsonenJ.; RuokolainenJ.; CeciP.Electrostatic Assembly of Binary Nanoparticle Superlattices Using Protein Cages. Nat. Nanotechnol.2013, 8, 52–562324165510.1038/nnano.2012.220.^1^*This work shows how virus and ferritin protein cages can be used to direct the self-assembly of binary nanoparticle crystals and how the electrolyte conditions govern the structural order*.
- Heuer-JungemannA.; LinkoV.Engineering Inorganic Materials with DNA Nanostructures. ACS Cent. Sci.2021, 7, 1969–19793496389010.1021/acscentsci.1c01272PMC8704036.^2^*This work summarizes diverse approaches for creating nanoparticle assemblies using DNA nanostructures as templates. It further adds to the topics presented here by describing versatile metallization and mineralization schemes for DNA frameworks*.
- Nonappa; LahtinenT.; HaatajaJ. S.; TeroT.-R.; HäkkinenH.; IkkalaO.Template-Free Supracolloidal Self-Assembly of Atomically Precise Gold Nanoclusters: From 2D Colloidal Crystals to Spherical Capsids. Angew. Chem., Int. Ed.2016, 55, 16035–1603810.1002/anie.20160903627879034.^3^*This work discloses how ligand engineering of atomically precise metal nanoclusters allows 2D assemblies by directed hydrogen bonding*.
- ZhangH.; ZengH.; PriimagiA.; IkkalaO.Programmable Responsive Hydrogels Inspired by Classical Conditioning Algorithm. Nat. Commun.2019, 10, 32673133219610.1038/s41467-019-11260-3PMC6646376.^4^*This work reported light-controlled linear self-assembly of gold nanoparticles in a gel network, utilized for the implementation of programmable responses inspired by classical conditioning*.

## Introduction

Self-assembly of colloidal particles for diverse functions^5^ requires narrow size dispersion, especially for hierarchical assemblies. This provides challenges for the synthetic materials science. In the biological world, evolution offers well-defined colloidal pathways for distinct physical and chemical properties.^6^ Understanding the structure–function relationships suggests biomimetic self-assembled materials.

Nanoparticles (NPs) with sufficiently narrow size distribution or atomically precisely defined nanoclusters (NCs) can self-assemble into 3D crystals,^7^ two-dimensional arrays,^8−13^ supraparticles,^14−18^ and colloidal capsids,^19^ allowing catalysis, enantioselective synthesis, sensing, drug encapsulation, and optoelectronics.^13,14,18,20^ To avoid packing problems due to NP size distributions, they can form capsulated hollow protein cages or viruses, which can direct the assembly independent of their composition.^21^ Furthermore, multicomponent cocrystals may be achieved.

On the other hand, programmable DNA-based systems^22^ suggest designs to encode precise nanoshapes and information processing operations.^23^ This is promoted by the robust DNA origami technique, where single-stranded DNA (ssDNA) molecules, called “staples”, fold into predefined 2D or 3D nanostructures through Watson–Crick base pairing.^24^ They are commonly megadalton-sized (dimensions <100 nm) yet are extendable to the gigadalton scale (dimensions >1 μm).^25^ Importantly, DNA origami enables accurate positioning of DNA-conjugated molecular components, even at sub-nanometer resolution.^26^

Atomically defined NPs or nanocrystals allow well-defined self-assemblies, especially through control over the ligand interactions. On the other hand, light enables remote and noninvasive control of NP assemblies,^27,28^ either by coupling light-responsive ligands to the NPs or by light-responsive molecules in the solution.^27−33^ Here we provide an account of our recent efforts on these approaches.

## Guided Assembly by Protein Cages

**Figure 1:**
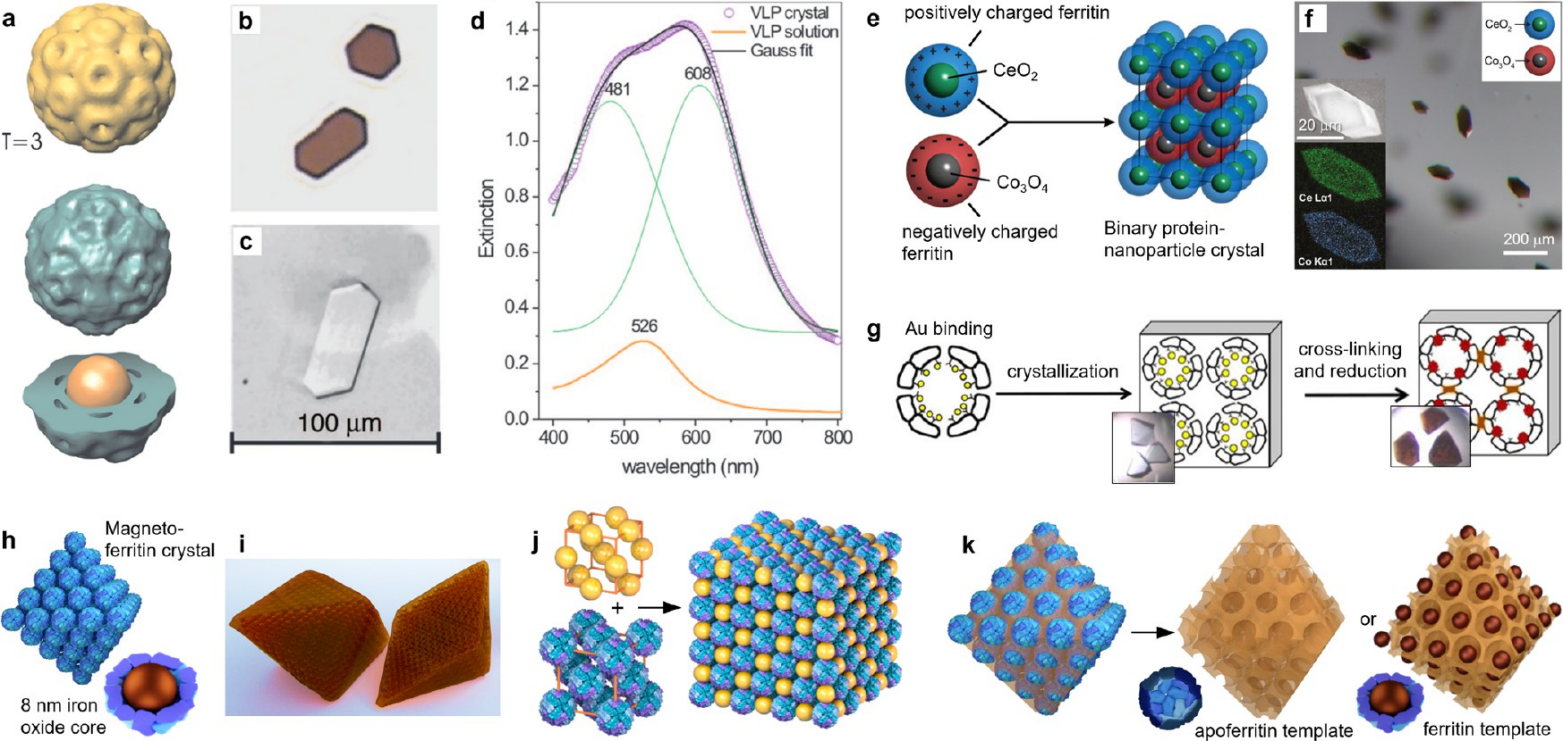
Precision nanoparticle assembly guided by protein cages. (a) TEM reconstruction of triangulation number *T* = 3 BMV–gold hybrid VLPs. (b) Transmission optical images of VLP and (c) BMV crystals. (d) Optical spectra of VLPs in crystal and solution. (e) Assembly mechanism and (f) microscopy characterization of binary ferritin crystals loaded with NPs. (g) Monitoring the nucleation and growth of gold clusters inside cross-linked apoferritin crystals. (h,i) Schematic images of magnetoferritin crystals. (j) Face-centered cubic (fcc) crystals formed by AuNPs and enzyme-loaded ferritin cages. (k) Mesoporous silica templated by (apo)ferritin crystals. Panels a–d reproduced with permission from ref (34). Copyright 2006 National Academy of Sciences. Panels e and f reproduced with permission from ref (35). Copyright 2016 American Chemical Society. Panel g reproduced with permission from ref (39). Published 2017 by Springer Nature Ltd. Panel j reproduced with permission from ref (44). Published 2019 by American Chemical Society. Panel k reproduced with permission from ref (45). Published 2021 by John Wiley & Sons.

An early demonstration of single crystals consisting of protein cages encapsulating NPs^34^ utilized the brome mosaic virus (BMV) capsid assembly over gold NPs (AuNPs), where the NP diameter controls the protein capsid size and structural symmetry. Using 12 nm AuNPs, triangulation number *T* = 3 capsids with a diameter of 29 nm assembled on the AuNP (Figure 1a). Crystals with dimensions up to ∼10 μm were obtained from the Au–BMV hybrid virus-like particles (VLPs) (Figure 1b) under the same conditions as the native virus (Figure 1c), indicating that the capsid directs the crystallization of the NP cargo. Optical characterization shows a red color and a spectrum exhibiting multipolar plasmonic coupling between the gold particles (Figure 1d).

The virus-guided approach was extended to genetically engineered oppositely charged ferritin cages,^35,36^ NP syntheses separately at both cages (Figure 1e), and electrostatic self-assembly for binary lattices with up to 150-μm edge length and a primitive tetragonal lattice (Figure 1f). Binary ferritin crystals consisting of both cerium oxide and cobalt oxide NPs were demonstrated, where the NP-loaded ferritin crystals possess the same structure as that formed by apoferritin (empty) cages. This demonstrates that the crystal lattice is determined by the protein. The binary crystal allows *in crystallo* catalysis, and for example, binary ferritin crystals loaded with cerium and iron oxide NPs show oxidase-like activity that can be sustained for several catalytic turnover cycles.^37^ Apoferritin crystals can also elucidate the mechanism of NP nucleation and growth.^38^ By loading Au ions into the crystal, followed by reduction with sodium borohydride, sub-NC formation was followed by X-ray analysis (Figure 1g).^39^

Protein crystallization has been applied to direct 3D arrays of magnetic NPs encapsulated inside ferritin cages (Figure 1h). The resulting crystals have a face-centered cubic (fcc) structure and octahedral habit with facet dimensions up to ∼100 μm (Figure 1i).^40^ The magnetostatic interactions between the encapsulated particles lead to collective behavior, such as low coercivity that is weakly dependent on the temperature.^41^ Furthermore, the blocking temperature depends on the hydration state of the crystal,^42^ and the hysteresis of the field-dependent magnetization is tunable by the crystallinity.^43^

The voids between the protein particles allow further functions. For example, magnetoferritin forms binary superlattices with cationic AuNPs toward interpenetrating simple cubic lattices that enhance the contrast in magnetic resonance imaging.^1^ Other examples include fcc lattices obtained from enzyme-loaded apoferritin and Au particles capable of artificial chaperone activity (Figure 1j).^44^ Inorganic silica matrices can be infiltrated into the free interstitial space between ferritin proteins to template mesoporous structures (Figure 1k). The crystal houses individual NPs in an “egg-carton” like manner in the pores created by protein removal during calcination.^45^

Protein-assisted NP crystallization is feasible to guide the phase behavior of colloidal particles and to build model systems for collectively acting materials. In principle, this could be achieved by modifying the sequences of constituent polypeptides and nucleic acids that play a crucial part in the formation of effective protein-based systems. However, developing the *de novo* design of such crystals remains a challenge. Intriguingly, recent advances in programmable DNA-based systems have enabled such modular frameworks.

## DNA-Guided Assembly

**Figure 2:**
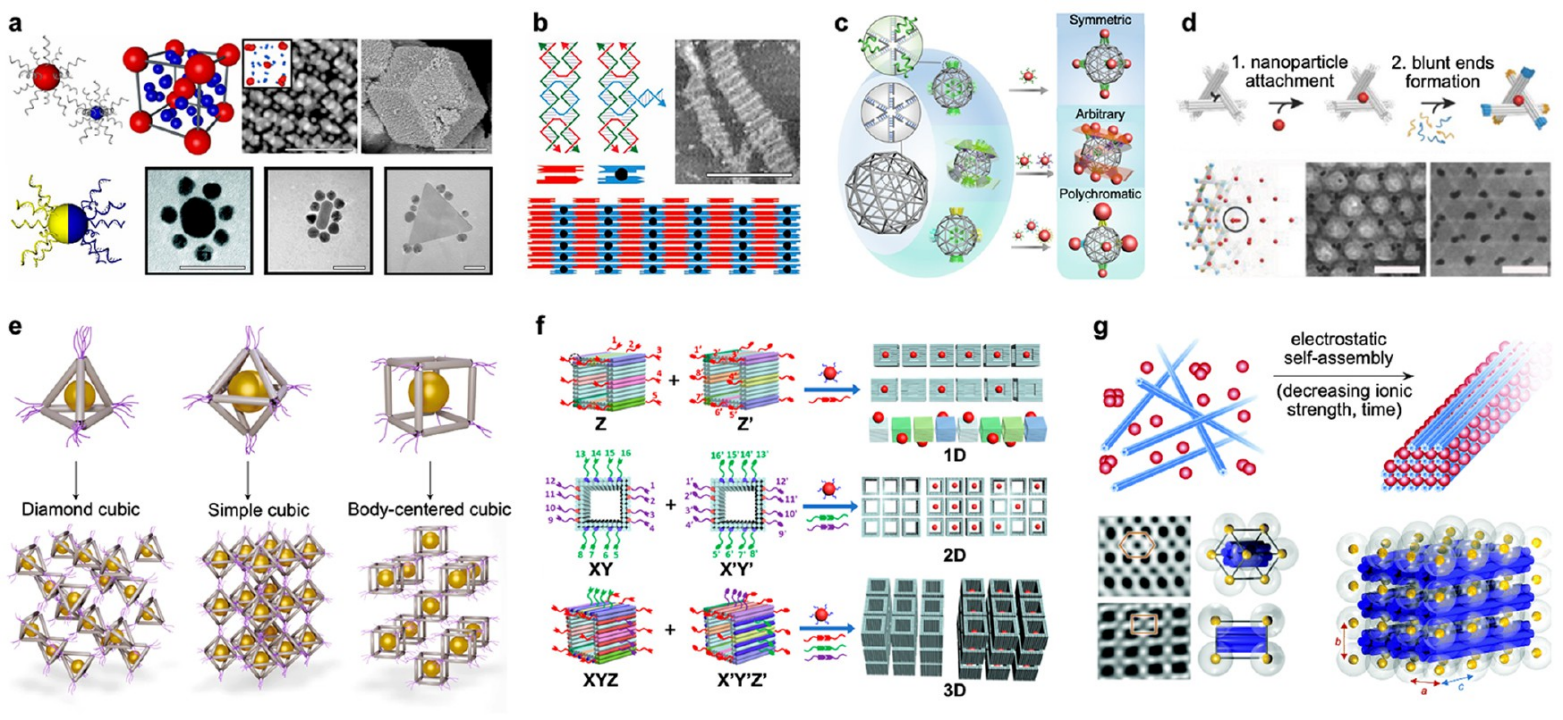
DNA-directed precision assembly of nanoparticles. (a) (top left) Spherical NPs capped with customized DNA sequences assemble into AB6-type superlattices. (top right) TEM image of the lattice with tomographic reconstruction as an inset (scale bar 100 nm) and (bottom right) SEM image of large faceted single crystals (scale bar 1 μm). (bottom left) Asymmetric distribution of surface-bound DNA strands enables core–satellite cluster formation. Scale bars in electron micrographs are 50 nm. (b) DNA double-crossover (DX) and DX+J tiles organize into a 2D lattice. The black dot depicts the protruding junction of the DX+J tile, acting as a binding site for NPs, for example. The scale bar is 300 nm. (c) Pentakis icosidodecahedron-shaped meshed DNA origami facilitates valence-programmable NP cluster architectures. The geometry and composition of the cluster is encoded using protruding ssDNA vertices and DNA-coated NPs. (d) Upon loading with AuNPs (step 1) and completing the struts with “polymerization strands” (step 2), DNA origami tensegrity triangles organize into a 3D rhombohedral crystalline lattice (bottom panel). TEM images show lattices with 10 and 20 nm NPs; the scale bars are 100 nm. (e) Metal NPs and DNA origami cages form material voxels (top panel). Tetrahedral (*v* = 4), octahedral (*v* = 6), and cubic (*v* = 8) voxels assemble into ordered lattices with valence-governed geometries. (f) Hollow DNA nanochambers are independently encoded along the orthogonal axes thus allowing for differentiated bonds. With programmable ssDNA-connector sets, various geometries and selective loading of the DNA voids are achieved. (g) (top) Oppositely charged AuNPs and 6HBs organize into a lattice through electrostatic interactions. (bottom) Inverse Fourier transforms calculated from TEM images along different projection axes of the lattice; hexagonal (top) and [100] (bottom) with schematic views and a 3 × 3 tetragonal unit cell. Panels a and b reproduced with permission from ref (46). Copyright 2015 AAAS. Panel c reproduced with permission from ref (48). Published 2020 by Springer Nature Ltd. Panel d reproduced with permission from ref (49). Copyright 2018 John Wiley & Sons. Panel e reproduced with permission from ref (50). Copyright 2020 Springer Nature Ltd. Panel f reproduced with permission from ref (51). Copyright 2020 American Chemical Society. Panel g reproduced with permission from ref (52). Published 2019 by Royal Society of Chemistry.

The addressability of DNA-based architectures enables engineering of various inorganic materials, from metallized or mineralized nanoshapes to crystals with defined configurations.^2^ DNA-directed particle assembly is governed by simple rules, that is, *valency*. DNA-assisted NP crystals form via two design principles: (1) NP-templated DNA bonds, where customized and flexible DNA sequences act as guiding surface ligands for the core particles (Figure 2a) or (2) hybridization-based DNA bonds, where higher-order systems assemble from DNA motifs and frameworks hosting different cargo (Figure 2b).^46^ They were pioneered by the groups of Mirkin (1) and Seeman (2), thus laying the foundation for DNA-programmable materials.^47^ In addition, valence-programmable NP clusters with various geometries may also form using a discrete DNA mesh frame origami as a templating core structure (Figure 2c).^48^

Seeman’s work with macroscopic 3D DNA crystals assembled from small DNA tensegrity triangles inspired lattices of DNA origami-based elements (Figure 2d).^49^ They were site-specifically equipped with 10 or 20 nm AuNPs (Figure 2d, step 1) and glued together through self-matching shape-complementary blunt ends of the struts (Figure 2d, step 2) resulting in rhombohedral AuNP lattices (Figure 2d, bottom panel).

DNA origami-directed nano-object assemblies were generalized by introducing valence-controlled material voxels (Figure 2e).^22,50^ The feasibility was demonstrated using tetrahedral (valence *v* = 4), octahedral (*v* = 6), and cubic (*v* = 8) DNA cages with ssDNA protrusions at each vertex. The DNA frameworks were loaded with proteins, metal NPs, and quantum dots as cargo. These DNA-prescribed material voxels were then annealed together through vertex-to-vertex hybridization. As the valence and coordination of the DNA cages unambiguously define the lattice geometry, 3D cubic diamond, simple cubic, and body-centered cubic crystals were produced.

An alternative path to programmable DNA-guided NP arrays was presented toward hollow DNA origami cuboids or “DNA nanochambers” (Figure 2f).^51^ Their exterior was functionalized with customizable multisequence strand sets that enabled addressable, directional, differentiated, and “polychromatic” bonds (Figure 2f, left). DNA cuboids with bonds encoded along their three orthogonal axes yielded heteropolymers, helical polymers, 2D lattices, and even mesoscale 3D nanostructures (Figure 2f, right). Through the adjustable binding modes and selective cargo-loading, this approach led to 1D, 2D, and 3D metal NP arrays (Figure 2f, right).

Complementary to the hybridization-derived strategies, DNA-assisted AuNP lattices may also be formed through solely electrostatic interactions between the crystalline-forming components.^52^ Therein, 2.5 nm AuNPs were employed with positively charged alkyl-oxyethylene ligands and, as the opposite-charged assembly counterparts, negatively charged six-helix bundle (6HB) DNA origami (Figure 2g, top). Upon lowering the ionic strength of the reaction buffer, the components organized into tetragonal superlattices (Figure 2g, bottom).

Therefore, precision NP placement within large ribosome-sized macromolecules can be achieved using DNA frameworks. Currently, it is possible to produce addressable DNA structures comprising millions of nucleotides^25^ and macroscopic DNA lattices containing ∼10^12^ DNA origami components.^53^ The ever-expanding dimensions of the DNA-based platforms and the foreseen integrated dynamicity of the assemblies^54,55^ open opportunities for optically transparent metamaterials, substrates, and devices, for example, with stimuli-triggered responses.^50^

## Self-Assembly of Precision Noble Metal Nanoparticles

**Figure 3:**
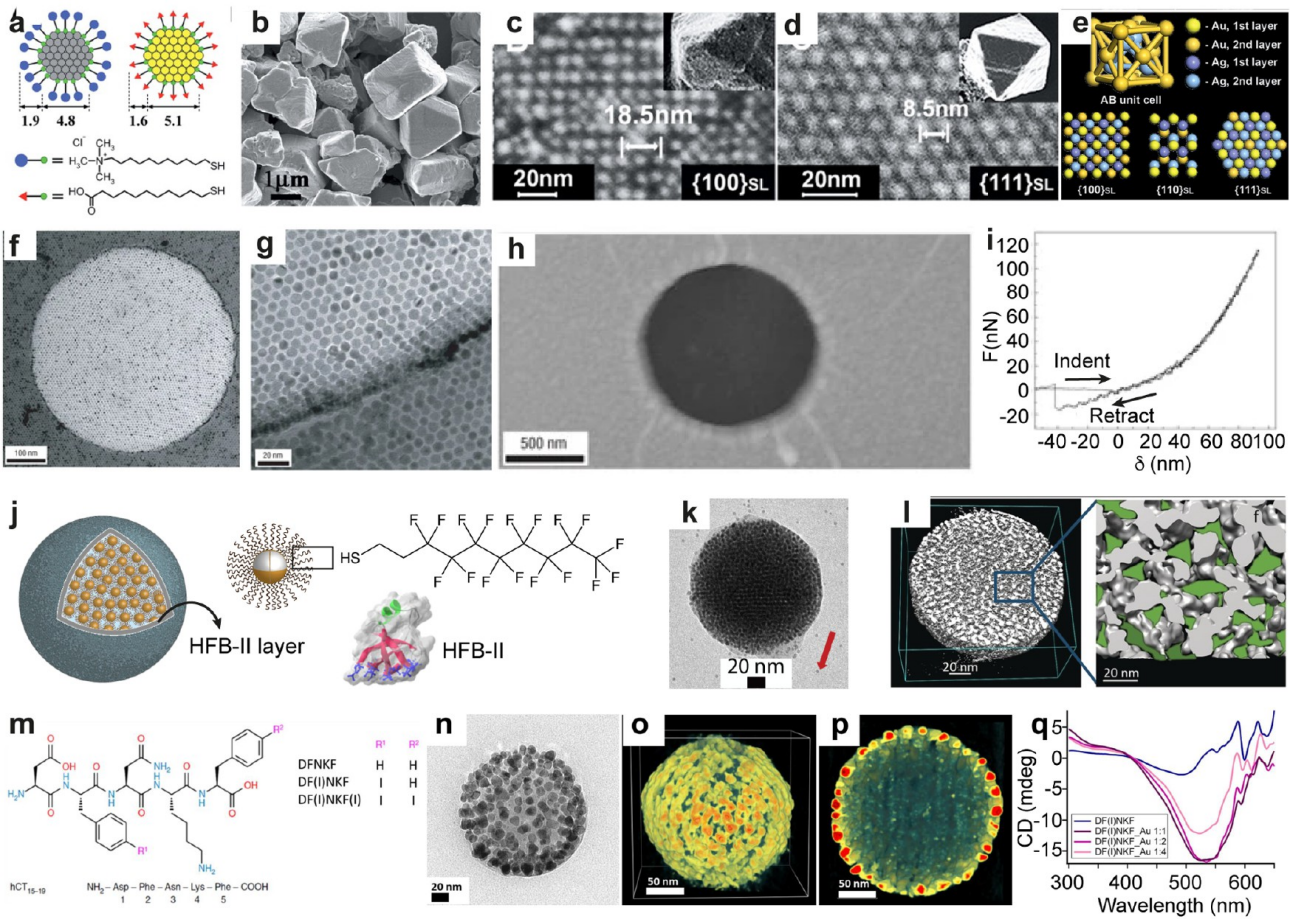
Self-assembled 3D crystals, 2D arrays, and spherical particles using narrow size dispersed nanoparticles. (a–e) Schematics of AuMUA and AgTMA and SEM, HRSEM, and scheme of diamond-like crystals with the projections of {100}_SL_, {110}_SL_, and {111}_SL_ planes, respectively. (f–i) TEM images, AFM image, and force vs. distance curve showing elasticity of the self-assembled dodecanethiol capped AuNP membrane. (j–l) Schematics, TEM image (red arrow indicates fiducial gold markers), and 3D-reconstructed electron tomogram, respectively, of fluorinated supraparticles assembled using HFB-II. (m) Chemical structure of iodinated peptides used for in situ NP formation. (n–p) TEM image, 3D-reconstructed tomogram, and cross-sectional view showing monolayer shell of AuNPs and unreacted peptides in the core, respectively. (q) CD spectra of DF(I)NKF with varying ratio of Au. Panels a–e reproduced with permission from ref (7). Copyright 2006 AAAS. Panels f–i reproduced with permission from ref (9). Copyright 2007 Springer Nature Ltd. Panels j–l reproduced with permission from ref (17). Copyright 2017 John Wiley & Sons. Panels m–q reproduced with permission from ref (18). Published 2019 by American Chemical Society.

Noble metal NPs with narrow size distribution and, more recently, atomically precise NCs allow self-assembly into one-, two-, and three-dimensional structural and functional assemblies.^56,57^ For example, sphalerite or diamond-like 3D-crystals using electrostatic self-assembly of oppositely charged narrowly size-dispersed NPs have been reported (Figure 3a–e).^7^ Therein, mercaptoundecanoic acid (MUA) capped AuNPs (AuMUA, *d* ≈ 5.1 nm) were mixed with 11-mercaptoundecyl-*N*,*N*,*N*-trimethylammonium chloride (TMA) capped silver NPs (AgTMA, *d* ≈ 4.8 nm). The crystallization depends on the particle size and size dispersity: Co-crystallization of narrowly size-dispersed oppositely charged NPs having the same metal core but surface-functionalized with either MUA or TMA, resulted in amorphous aggregates or poor crystals. Interestingly, oppositely charged particles with different size distribution (e.g., AgTMA and AuMUA) resulted in high quality crystals. In another approach, self-assembly of like-charged NPs into faceted 3D crystals was demonstrated.^58^

Such examples demonstrate the effect of NP size distribution to control electrostatic self-assembly into 3D crystals. By contrast, NPs functionalized with hydrogen bonding (H-bonding) ligands offer better control due to their directionality. DNA- and other H-bonding functionalized NPs self-assemble into 3D crystals by tuning the melting temperature.^59−61^

Two-dimensional (2D) assembly for binary superlattice and NP membranes proceeds using evaporation-induced assembly, Langmuir–Blodgett method, and DNA-directed NP assembly.^8−13^ For example, 2D arrays of NPs were reported using the evaporation-induced assembly of dodecanethiol ligand capped AuNPs with an average diameter of ∼9.4 nm (Figure 3f–i).^9^ The membranes display Young’s modulus of several gigapascals (Figure 3i).

Beyond the 3D crystals and 2D close packed arrays, tailored NPs achieve supraparticles,^14−18^ reversible capsids,^19^ and chiroptically active spherical superstructures.^20^ Fluorophobic-driven assembly of narrowly dispersed AuNPs into supraparticles was shown (Figure 3j–l).^19^ AuNPs with bimodal distributions of 1.6 ± 0.6 nm and 3.8 ± 0.8 nm were stabilized by 1*H*,1*H*,2*H*,2*H*-perfluorodecanethiol and self-assembled into spherical superstructures (*d* ≈ 50–200 nm) in the presence of hydrophobin II (HFB-II) protein. The HFB-II formed a protective shell around the superstructure (Figure 3j). The fluorinated ligands offered confined space between the NPs (Figure 3l) for encapsulation of poorly water-soluble fluorinated drugs (Figure 3l). In another approach, chiroptically active AuNP supraparticles (Figure 3n–p) were achieved using a peptide-mediated *in situ* gold reduction with modified human calcitonin-derived amyloidogenic peptides (Figure 3m).^18^ The initial Au(III)–peptide assemblies underwent spontaneous reduction on the surface of the superstructures resulting in Au(I), which acted as a source of iodide ion (I^–^) and Au(0). The use of 2,2′-bipyridine as a scavenger for Au(I) prevented the formation of AuNPs. A strong CD signal suggested that the supraparticles are chiroptically active (Figure 3q).

Even though some control over shape and stability was shown, monodispersity remained challenging. Therein, atomically precise AuNCs (*d* < 3 nm) opened new opportunities.^62−68^ They contain exact numbers of metal atoms and surface ligands. Their stability is controlled by ligand passivation using small molecules, synthetic polymers, or biomacromolecules, similar to plasmonic NPs. Their small size, well-defined surface functionalities, and dispersion behavior provide multiple advantages. Higher-order assemblies were achieved using air–water interface, solvent evaporation, and solution-based assembly.^68^ Assembly of Au_55_(PPh_3_)_12_Cl_6_ (PPh_3_ = triphenylphosphine) was reported using the solvent evaporation method toward a fcc lattice.^69^ In another approach, electrostatic assembly of [Au_55_(Ph_2_PC_6_H_4_SO_3_H)_12_Cl_6_] was used in two-dimensional monolayers of clusters on polyethylenimine coated TEM grids.^70^ A modified Langmuir–Blodgett technique allows quasi-one-dimensional stripes of Au_55_(PPh_3_)_12_Cl_6_ NCs.^71^

**Figure 4:**
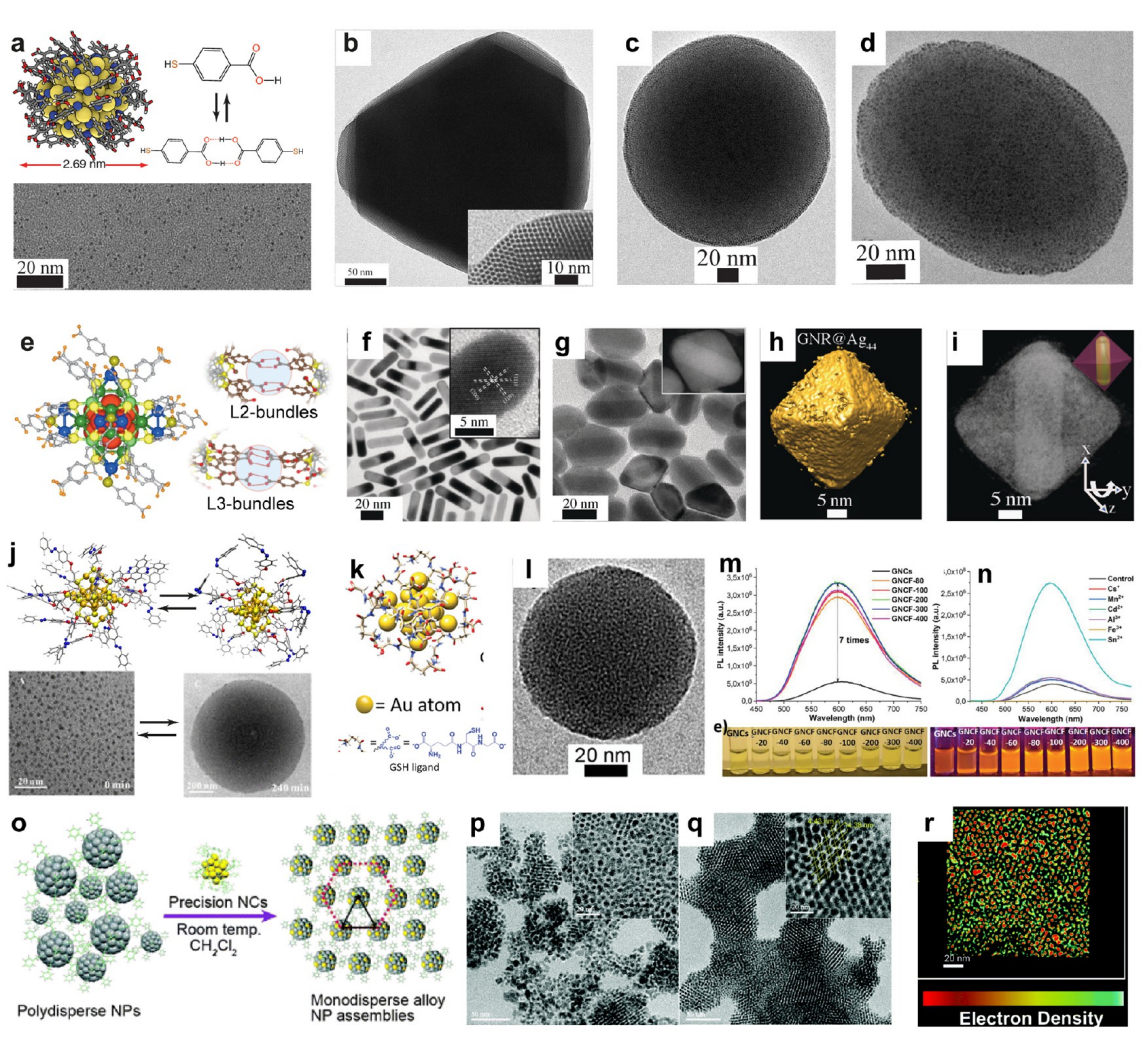
Self-assembly of precision gold and silver nanoparticles. (a–d) Single crystal X-ray structure of Au_102_–*p*MBA_44_, TEM images of 2D colloidal crystal (hcp arrays in the inset), spherical, and ellipsoidal capsids, respectively. (e) X-ray structure of Na_4_Ag_44_*p*MBA_30_ and representation of H-bonding ligand bundles. (f–i) TEM images of *p*MBA functionalized GNRs and GNR–Ag_44_ composite nanocages, STEM tomography, and a cross-sectional view of a single composite cage. (j) *Cis*–*trans* isomerization of azobenzene capped Au_25_ NCs and TEM image of light triggered reversible assemblies. (k, l) Schematics of glutathione (GSH) capped gold NC and TEM image of a AuNC framework (GNCF). (m, n) PL intensity of GNCs as a function of Sn^2+^ and different divalent ion concentrations, respectively. (o) Schematic representation of reaction between the polydisperse NPs and precision NCs, (p–r) TEM images of polydispersed NPs before the reaction and monodisperse alloy NPs and a cross-sectional view of a 2D assembly of alloy NPs, respectively. Panels a–d reproduced with permission from refs (3) and (75). Copyright 2016 and 2018 John Wiley & Sons. Panel e reproduced with permission from ref (76). Copyright 2013 Nature Publishing Group. Panels f–i reproduced with permission from ref (79). Copyright 2018 John Wiley & Sons. Panel j reproduced with permission from ref (85). Copyright 2020 American Chemical Society. Panels k–n reproduced with permission from ref (84). Copyright 2019 John Wiley & Sons. Panels o–r reproduced with permission from ref (89). Copyright 2020 Royal Society of Chemistry.

However, triphenylphosphine protected clusters suffer from stability problems. The Brust-synthesis using thiolated ligands has opened new avenues for stable atomically precise NCs.^72^ The crystal structure of the water-soluble Au_102_–*p*MBA_44_ NC (*p*MBA = *para*-mercaptobenzoic acid) has been disclosed.^73^ Its crystallization in the centrosymmetric space group *C*2/*c* suggests gold–thiol interaction, ligand orientation, and patchy ligand distribution.^74^ Subsequently, solution-based self-assembly of Au_102_–*p*MBA_44_ NCs into 2D colloidal crystals and spherical capsids was reported (Figure 4a–d).^3,75^ While most 2D crystals are prepared using molecular precursors under a growth control mechanism, this approach utilizes symmetry breaking due to the anisotropic ligand distribution. Au_102_–*p*MBA_44_ is soluble in methanol when all the carboxylic acid groups are protonated. However, upon partial deprotonation of a certain number of COOH groups (∼22), the clusters are water-dispersible but insoluble in methanol. Importantly, the deprotonation is not random; instead, it creates a patchy distribution of protonated and deprotonated units, revealing anisotropic distribution of H-bonding COOH dimerization around the cluster surface. Therefore, anisotropic structure was realized upon a combination of electrostatic repulsion and H-bonding between the NCs.

The slow exchange of methanol with water through dialysis resulted in 2D nanosheets with hexagonal close packing and interparticle distance of 2.7 nm. This distance is close to the overall size of the NC (2.69 nm), suggesting that they interact via stacked supramolecular interactions. By contrast, rapid addition of an aqueous solution to methanol resulted in spherical and ellipsoidal superstructures, termed capsids, with monolayer thick shells (Figure 4c,d). This is attributed to the packing defects due to a rapid assembly, inducing bending. Such structures suggest lightweight porous colloidal framework materials. The concept was extended to other materials and synergistic interactions such as combinations of H-bonding and magnetic field-induced dipolar interactions.^19^

Ultrastable AgNC Na_4_Ag_44_*p*MBA_30_ allows rhomboid crystals where the carboxylic acid groups in fully protonated form provide a H-bonded network due to their patchy ligand bundles (Figure 4e).^76^ The bundles of three ligands (L3) promote strong intralayer H-bonding, whereas the bundles of two ligands (L2) promote interlayer H-bonding.^77^ Na_4_Ag_44_*p*MBA_30_ allows telluride nanowire (TeNW) bilayers by decorating NCs on the TeNW surface.^78^ Gold nanorod (GNR)–Ag_44_ (*d* ≈ 10 nm, *l* ≈ 30 nm) (Figure 4f) functionalized with *p*MBA interacts with Na_4_Ag_44_*p*MBA_30_ in *N*,*N*-dimethylformamide, resulting in selective growth of octahedral cages and encapsulation of GNRs within the cage (Figure 4g–i).^79^ Each cage contains a single nanorod, where the individual components preserve their identities. The absorption spectrum of the composite cages displays peaks arising from GNRs as well as the NCs. A significant broadening and shift in peak position in the NIR region were observed for the composite material, thus suggesting electronic interaction between the GNR and NCs. Even though Na_4_Ag_44_*p*MBA_30_ crystallizes in a triclinic lattice, based on computational simulations, the lattice structure of octahedral assemblies is fcc. When water-soluble *p*MBA-functionalized Au_102_ or Au_250_ NCs are used, composite structures containing a monolayer shell of NCs around a GNR form,^79^ as AuNCs require partial deprotonation in an aqueous medium to disperse. Therein, only a limited number of H-bonding groups are available, and the electrostatic repulsion stabilizes the individual structures.

Another subtlety of gold and silver NCs deals with luminescence.^80−83^ Despite their high photothermal stability, NCs typically display low quantum yields. However, a metal coordination route showed simultaneously self-assembled framework structures and enhanced photoluminescence quantum yield (Figure 4k–n).^84^ Glutathione (GSH) capped AuNCs, Au_25_(SG)_*n*_, were treated with various divalent metal ions. In the presence of Sn^2+^, highly tunable AuNC framework structures (GNCFs) are obtained. The quantum yield was increased from 3.5% for individual clusters to 25% for the frameworks. The 3D architecture of the GNCFs enhanced the adsorption of dye molecules and increased photocatalytic activities by 20-fold compared to individual NCs. Notably, the GNCFs displayed higher cell viability and cellular uptake than NCs when tested using NIH3T3 and A549 cells. This is attributed to the fact that individual clusters may generate reactive oxygen species and undergo uncontrolled aggregation, causing cell death.

Toward fully reversible supracolloidal assembly of NCs, light-triggered reversible assembly of Au_25_ NCs stapled with azobenzene-alkyl monothiol (C_3_-AMT), that is, [Au_25_(C_3_-AMT)_18_]^−^, into colloidal disc-like superstructures was explored (Figure 4j).^85^ Irradiation in dichloromethane with ultraviolet light resulted in visible color change, and the TEM imaging suggested disc-like stable supracolloidal structures (*d* ≈ 100–1000 nm). However, upon irradiation with visible light, the structures dispersed into individual NCs.

The molecular nature of NCs offers selective doping, inter-NC reactions, isotopic exchange between the clusters, and cluster–NP reaction.^86−88^ Atom transfer reactions have been shown between atomically precise 2-phenylethanethiol (PET) capped Au_25_(PET)_18_ NCs and PET protected polydispersed AgNPs (*d* ≈ 4 nm).^89^ The reaction resulted in monodisperse alloy NPs, which spontaneously assembled into 2D superstructures (Figure 4o–r). The reaction was ligand-specific, and the kinetics depended on the size dispersities of the AgNPs.

## From Light-Controlled Self-Assembly to Logic Gates

**Figure 5:**
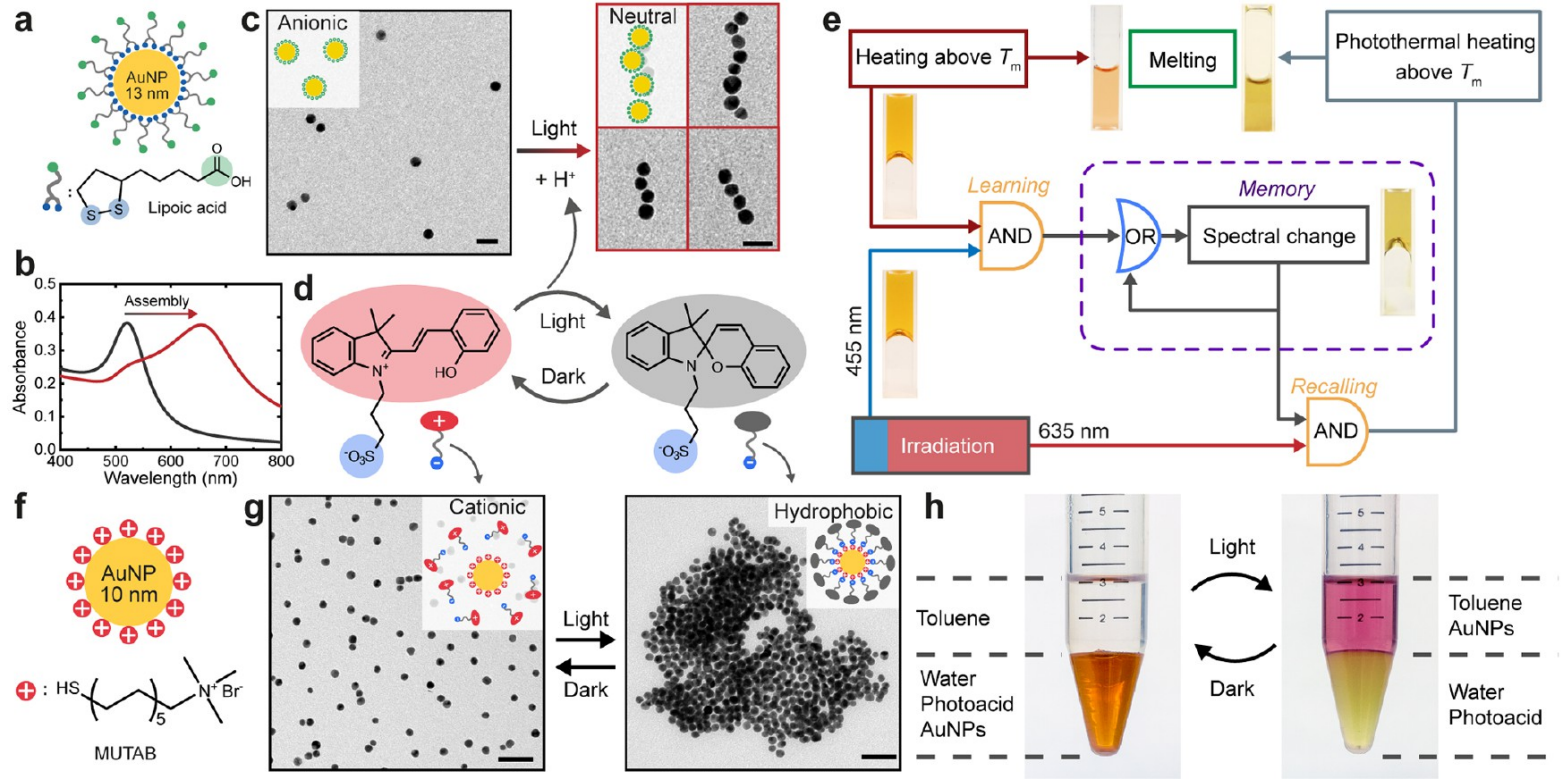
(a) AuNPs modified with lipoic acid (LA-AuNPs). (b) UV–vis spectra of dispersed (black) and assembled (red) LA-AuNPs. (c) TEM images showing the linear self-assembly of LA-AuNPs induced by light-triggered pH change. Scale bars 30 nm. (d) Illustration of the light induced isomerization of the photoacid. Upon irradiation, a proton is released, accompanied by the zwitterionic–anionic transition of the photoacid molecule. (e) Logic diagram of the response in an agarose network containing LA-AuNPs and photoacid, which shows classical conditioning behavior in response to light and heat. (f) Cationic AuNPs modified with (11-mercaptoundecyl)-*N*,*N*,*N*-trimethylammonium bromide (MUTAB). (g) TEM images showing the light-induced reversible hydrophobization of the cationic AuNPs due to adsorption/desorption of the photoacid. Scale bars 50 nm. (h) Photographs showing reversible transfer of cationic AuNPs between water and toluene. Panels a–c and e reproduced with permission from ref (4). Published 2019 by Springer Nature Ltd. Panels d and f–h reproduced with permission from ref (90). Copyright 2019 Royal Society of Chemistry.

Among approaches to control the self-assembly of NPs, light is particularly versatile.^27,28^ This can be achieved by either coupling light-responsive ligands directly to NPs or having dissolved light-responsive molecules that cause changes on the surface of NPs.^29−33^ Herein, we discuss two examples using a dissolved merocyanine-based photoacid to control the assembly of modified AuNPs (Figure 5).^4,90^ Typically, the light-triggered assembly of NPs results in large (>100 NPs) and irregular aggregates.^27,28^ Therefore, the plasmonic coupling between the NPs is not well-controlled. By choice of a pH-responsive ligands for AuNPs (lipoic acid, LA), linear assembly has been achieved with light via the photoacid (Figure 5a–e).^4^ Therein (Figure 5c), a strong plasmonic band appears above 650 nm compared to the original 520 nm of dispersed AuNPs (Figure 5b). Such assembly results from the competition of attractive H-bonding and van der Waals interactions and repulsive electrostatic interactions.^91^ In this kinetically controlled process, the released protons from the photoacid upon irradiation partially neutralizes the negative charges on the AuNPs, leading to formation of AuNP dimers at the start of random aggregation. As the electrostatic repulsion becomes asymmetric on the dimers and subsequent oligomers, linear AuNP chains grow as the newly assembled AuNPs preferably attach to the end of the chain instead of its side. Such assemblies are stable and do not spontaneously disassemble in the dark, unless additional bases are added to fully deprotonate the carboxylic groups at pH > 8.

Therein, a programmable hydrogel has been achieved, consisting of agarose, photoacid, and LA-AuNPs.^4^ The hydrogel shows responses algorithmically inspired by classical conditioning, that is, a simple form of associative learning (Figure 5e).^4^ Herein, the hydrogel intrinsically responds to heating by the gel–sol transition (melting) and does not respond to 455 and 635 nm light irradiation due to the low absorbance. Upon conditioning, the AuNPs undergo light-induced self-assembly to form chains when exposed simultaneously to heating and irradiation. This leads to significantly increased absorption at 635 nm, thus switching on the “memory” of the hydrogel. Subsequently, the conditioned gel can melt upon irradiation alone due to the enhanced photothermal effect, causing a temperature increase above the melting point of the agarose. The conditioning process thus involves the association of a neutral stimulus (light) with an unconditioned stimulus (heat), and the self-assembly of LA-AuNPs serves a central role by acting as the memory module. Importantly, the AuNPs in agarose do not assemble upon irradiation alone, as diffusion is hindered by the gel network. Furthermore, clock reactions with temporally controlled pH profiles can be incorporated to allow programmed forgetting and spontaneous memory recovery, inspired by the biological systems.

On the other hand, an intriguing but often neglected fact is that the charge state of the photoacid itself changes due to the loss of proton during the photoisomerization process.^90^ The photoacid in Figure 5d switches between zwitterionic and anionic forms when in the dark and under irradiation, respectively. Therefore, the photoacid allows triggering of the assembly of non-pH-responsive NPs by adsorption of the photoacid molecules (Figure 5f–h). The cationic AuNPs are stable in the aqueous photoacid solution in dark, as the photoacid in the zwitterionic form lacks strong interactions with the AuNPs. Upon irradiation, the photoacid switches to the anionic spiropyran form and adsorbs electrostatically onto the cationic surface of the AuNPs. Consequently, the surface charges are neutralized, and the AuNPs become hydrophobic. This leads to the assembly of the AuNPs into large aggregates (Figure 5g). The assembly is highly reversible, unlike the case of LA-AuNPs, due to efficient desorption of the photoacid and the high zeta potential of the cationic AuNPs. The kinetics of the disassembly can be controlled by temperature, resulting in an assembly lifetime in the dark between 10 s and 20 min as the temperature changes between 15 and 50 °C. Notably, the hydrophobization strategy allows quantitative and reversible transfer of the cationic AuNPs between water and a nonpolar solvent such as toluene (Figure 5h), which may be utilized for purification or selective catalysis. Furthermore, the process can also be applied on macroscopic scale, demonstrated by the contact angle change from 0 to 60° of a photoacid droplet on a cationic surface upon irradiation.

## Implications

Synthetic colloidal particles are typically polydispersed, thus hindering long-range self-assembled order. Two main routes can be conceptualized to tackle the problem by guidance from biological motifs. Long-range order of synthetic colloids can be obtained by trapping them in precisely defined protein cages. Nevertheless, although *de novo* design of atomically precise proteins has achieved an extremely sophisticated level,^92^ colloidal assemblies can also be guided by DNA nanotechnology allowing customizable sub-nanometer patterning resolution. It has been shown that the magnitude of the solution-phase fluctuations of DNA domains in a compact DNA origami nanostructure are similar to that of proteins.^93^ As the amino acid sequence determines the protein size, shape, and function, DNA sequences analogously encode accurate DNA nanostructures. Overall, user-defined DNA nanostructure design and assembly do not require massive computational power or experience in protein synthesis, and therefore DNA nanotechnology may, in a straightforward manner, open avenues, for example, for optically transparent metamaterials, substrates, and devices with stimuli-triggered responses. On the other hand, routes for narrow size distribution NPs and even precisely defined synthetic NCs have recently emerged for high structural control, thus providing new implementations.

Finally, we discuss responsive colloids for advanced functions. Life is the ultimate inspiration for materials science. To go beyond the classic stimuli-responsive materials, algorithmic mimics of elementary learning processes are needed for the true “intelligent” materials of the future. We disclose how triggerable plasmonic memories of NPs allow for responsive functions, inspired by the classical conditioning of psychology. We foresee numerous possibilities in interfacing materials science with biology, either structurally or algorithmically, providing guidelines for designing new functions.
